# Evaluation Resources for Asthma Programs and Beyond

**DOI:** 10.5888/pcd21.240035

**Published:** 2024-08-01

**Authors:** Samuel Dunklin, Sarah Gill, Maureen Wilce

**Affiliations:** 1 Centers for Disease Control and Prevention, National Center for Environmental Health, Division of Environmental Health Science and Practice, Asthma and Air Quality Branch, Atlanta, Georgia

## Abstract

Evaluation can ensure the quality of public health programs. Systematic efforts to identify and fully engage everyone involved with or affected by a program can provide critical information about asthma programs and the broader environment in which they operate. To assist evaluators working at programs funded by the Centers for Disease Control and Prevention (CDC’s) National Asthma Control Program (NACP), we developed a package of tools that build on the CDC’s 1999 Framework for Program Evaluation in Public Health. The resulting suite of evaluation tools guides evaluators through a structured but flexible process, engaging a diverse array of interest holders and actively involving them in evaluation planning and implementation, all while strengthening their capacity to meaningfully contribute to the evaluation process. For our newest tool, our team reviewed the recent evaluation literature to create an enhanced version of the 1999 framework that describes important elements of professional evaluation practice. Although the original framework describes the steps to take in conducting an evaluation and the standards for a high-quality evaluation, our enhanced framework includes an explanation of how evaluators should approach their work: by incorporating critical reflection, interpersonal competence, situational awareness, and cultural responsiveness. In this article, we highlight many of the evaluation resources our team has created since the NACP’s inception, culminating in a free e-text called *Planting the Seeds of High-Quality Program Evaluation in Public Health.* Public health professionals working in many types of programs — not just asthma — may find these resources useful.

**Summary**

**What is already known on this topic?**

Although program evaluation is central to public health, many professionals are untrained in this practice.

**What is added by this report?**

We summarize the work of the National Asthma Control Program’s evaluation team and its wide selection of tools. These tools are publicly available and useful for a wide array of public health professionals, extending beyond the asthma field.

**What are the implications for public health practice?**

Tools described support evaluation capacity building and the distribution of foundational evaluation tools.

## Background

Evaluation, the “process of determining the merit, worth, or value of something, or the product of that process” (1), is central to public health practice and is an essential service of public health (2). It is a means for asking and answering important questions about how we can improve the public’s health and be accountable for public funds. Evaluation is also included in several public health professional competency sets (3,4). Accordingly, the demand for evaluation is high, especially among agencies within the US Department of Health and Human Services, where evaluation has become a tool that public health practitioners and their partners use to make evidence-informed decisions (5).

Despite the importance of evaluation, research studies suggest that many public health professionals who enter the field through formal academic routes (eg, master’s degree in public health from accredited institutions) may graduate without completing a course in evaluation (6,7). Post graduation, the availability of professional development opportunities and funds to support them is limited. As a result, public health agencies and organizations often do not have ready access to staff with the skills and knowledge needed to competently plan and conduct evaluations or to commission and monitor evaluation contracts. It is this gap that we, evaluators in the Centers for Disease Control and Prevention’s (CDC’s) National Asthma Control Program (NACP), set out to fill, both through tailored technical assistance for our partners and the creation of a variety of resources.

As NACP celebrates 25 years of public service, we recognize the importance of documenting what has been accomplished during those years. This article is not only a historical account of the evaluation team’s time in NACP, but also a reflection on a selection of the publicly available tools and materials that have been developed. We hope that this article will serve as one of those tools to further support the work that you perform or give you a moment’s pause while you discover something new.

## Evaluation as a Tool for Systematically Learning How to Improve Programs

Evaluation conducted in keeping with professional standards is a responsive and collaborative undertaking. All 4 of what we consider foundational documents for the evaluation field call on evaluators to engage with interest holders to guide their work. These are the American Evaluation Association's (AEA) Guiding Principles, the Program Evaluation Standards (created by the Joint Committee on Standards for Education Evaluation and used broadly in the field to define standards for high-quality evaluation), AEA’s Public Statement on Cultural Competence, and the AEA Evaluator Competencies (8–11). In developing evaluation capacity-building materials for its funded partners, the NACP relied heavily on these documents and mirrored their collaborative approach.

NACP partners include the staffs of state, local, and territorial health departments and their many partners as well as staffs of national nongovernment organizations. Staff members responsible for evaluation activities in these organizations have varying backgrounds and levels of evaluation experience and expertise. Many come with epidemiology or research backgrounds and bring some of the technical skills required for evaluations, but they are often unfamiliar with the context in which those skills are to be applied.

In NACP’s early days, few resources were available to respond to partners’ requests for guidance on what was often, for them, a new responsibility. A new tool at the time was CDC’s Framework for Program Evaluation in Public Health (CDC Framework) (12). This framework set forth a flexible 6-step process for evaluating all aspects of public health programming. The framework emphasized the inclusion of interest holders — people affected by the program or its evaluation — and the importance of using an evaluation’s processes and findings to guide program improvement. Partly because of its apparent simplicity, the graphic depicting the evaluation steps and standards became an icon for many public health professionals. Even so, partners reported that they had difficulty applying the framework.

In response, NACP hired a team of evaluators to help build evaluation capacity internally and in the asthma programs it funded. Initially, staff members delivered introductory trainings focused on demystifying evaluation (13). As partners expanded the scope of their evaluation activities, we recognized a need for more comprehensive capacity building. The evaluation team tapped NACP program staff members who had an interest in evaluation to help tailor our approach to the programs’ needs, and we started to build out a suite of resources. Over the ensuing decade and a half, we created the tools to build these resources (Table 1). As you read, we encourage you to imagine how you might use evaluation generally and these tools specifically to demonstrate your program’s value and identify ways to improve your impact on the public’s health.

**Table 1:** Inventory of Evaluation Tools Developed by CDC’s National Asthma Control Program (NACP)^a^

Tool	Description and Use
Learning and Growing Through Evaluation: State Asthma Program Evaluation. https://www.cdc.gov/national-asthma-control-program/php/program_eval/guide.html	A series of 6 modules that provide step-by-step instructions and tools to facilitate the entire evaluation process. The modules help people responsible for leading and participating in evaluations build evaluation capacity and assure findings will be useful.
Module 1: Planning Evaluations. https://www.cdc.gov/national-asthma-control-program/php/program_eval/eval_guide/AsthmaProgramGuide_Mod1.pdf	Focuses on planning evaluations. Includes guidance on and templates for individual evaluation plans as well as a multi-year strategic evaluation plans. Select other tools and templates include: Program Activity Profile Potential Criteria for Evaluation Prioritization Evaluation Question Development Table Evaluation Design and Data Collection Summary Table
Module 2: Implementing Evaluations. https://www.cdc.gov/national-asthma-control-program/php/program_eval/eval_guide/AsthmaProgramGuide_Mod2_1.pdf	Provides users with a myriad of strategies to assist in the successful implementation of an evaluation. Includes tools and appendixes with detailed information on: Ways to Work with Interest holders Checklist for Successful Implementation of an Individual Evaluation Plan Meeting Evaluation Challenges Evaluation Management Toolkit Budgeting for Evaluation Developing an Action Plan
Module 3: Evaluating Partnerships. https://www.cdc.gov/national-asthma-control-program/php/program_eval/eval_guide/AsthmaProgramGuide_Mod3.pdf	This module focuses on the specific challenges that come with assessing collaborations. It includes tools and appendixes, including: Partnership Concept Map for the NACP Evidence Base on Effective Partnerships Crosswalk of Partnership Concepts with Sample Evaluation Questions and Tools Health Equity and Evaluation Potential Practices for Incorporating Equity into Partnership Evaluation
Module 4: Evaluating Asthma Surveillance. https://www.cdc.gov/national-asthma-control-program/php/program_eval/eval_guide/AsthmaProgramGuide_Mod4.pdf	Based on CDC’s Updated Guidelines for Evaluating Public Health Surveillance Systems, this module tailors the evaluation process for asthma surveillance systems. It includes: Sample Surveillance Evaluation Questions Sample Criteria of Merit and Indicators for Asthma Surveillance Evaluations Example of Indicators and Associated Performance Standards
Module 5: Evaluating Services and Systems Interventions. https://www.cdc.gov/national-asthma-control-program/php/program_eval/eval_guide/asthmaprogramguide_mod5.pdf	This module focuses on evaluation of coordinated activities designed to achieve outcomes at the individual or population level. Tools included address: Overarching intervention evaluation question types — process question Overarching intervention evaluation question types — outcomes questions Evidence continuum and types of evaluation Using social science theory in evaluation Relationship of logic model elements, evaluation questions, criteria of merit, and indicators
Module 6: Economic Evaluation for Asthma Programs. https://www.cdc.gov/national-asthma-control-program/php/program_eval/eval_guide/asthmaprogramguide_mod6.pdf	This module shows how to add economic evaluation to an overall evaluation portfolio. It includes: Potential Interest Holders by Evaluation Perspective Commonly Used Analytic Methods in Economic Evaluation Analytic Methods and Associated Summary Measures Distinguishing Characteristics of Economic Evaluation Components of Resources Consumed and Outcomes Realized in a Public Health Programs Templates for Managing Cost Data
Evaluator Self-assessment. https://www.cdc.gov/evaluation/tools/self_assessment/index.htm	This tool encourages evaluators to systematically reflect on and inquire about their own capacity to conduct high-quality program evaluations. Users can identify professional development needs and strengths to further develop. [Originally developed by NACP, CDC’s Office of Policy, Performance and Evaluation now hosts this tool.]
Good Evaluation Questions Checklist. https://www.cdc.gov/national-asthma-control-program/media/pdfs/2024/05/AssessingEvaluationQuestionChecklist.pdf	Based on the program evaluation standards, the checklist facilitates discussions among interest holders to assure that the evaluation questions selected for an evaluation are appropriate to guide the evaluation. It also serves to document the rationale and process for selecting questions.
Program Evaluation Tip Sheet: Integrating Cultural Competence into Evaluation: https://www.cdc.gov/national-asthma-control-program/media/pdfs/2024/05/cultural_competence_tip_sheet.pdf	This guide and tip sheet enable user to respond to persistent disparities in health outcomes with sensitivity and flexibility and work effectively in diverse contexts. These tools apply the program evaluation standards to highlight opportunities for integrating cultural competence throughout the six steps of the CDC Framework for Program Evaluation.
Cultural Competence Assessment Tool for State Asthma Programs and Partners (CCAT). https://www.cdc.gov/national-asthma-control-program/media/pdfs/2024/05/CCAT.pdf	The CCAT is a practical resource designed to promote and enhance cultural competence among partner organizations. Based on the Culturally and Linguistically Appropriate Service (CLAS) Standards, the CCAT is a self-assessment tool designed to assist programs in assessing the cultural competence of their own programs. Using a flexible, team-based approach, programs use the CCAT internally, with the aim of identifying program strengths and areas for improvement in cultural competence.
Practical Evaluation Using the CDC Evaluation Framework — A Webinar Series for Asthma and Other Public Health Programs. https://www.cdc.gov/national-asthma-control-program/php/program_eval/webinars.html	Nationally recognized experts present a general introduction to program evaluation; note challenges in conducting useful evaluations as well as methods for overcoming those challenges; and introduce the 6 steps of the CDC Framework for Program Evaluation. Webinars range from 15–65 min; PDFs of slides and scripts are posted.
Learning & Growing through Evaluation: Modules 1-6. https://www.cdc.gov/national-asthma-control-program/php/program_eval/guide.html	These documents highlight real world examples of how asthma programs have improved their programs with evaluation. Each entry describes the program or activity being evaluated, how the evaluation team conducted the evaluation, what the program learned during the evaluation, and how the program improved by using the results of the evaluation.
Planting the Seeds of High-Quality Program Evaluation in Public Health. https://www.cdc.gov/asthma/program_eval/PlantingSeeds_eTextbook-508.pdf	This free evaluation e-textbook is designed for public health professionals responsible for evaluation activities and for public health students. It is suitable for use in undergraduate and graduate public health programs and includes an overview of evaluation theory as well as practical tools and templates. It also provides an enhanced version of CDC’s evaluation framework that emphasizes the importance of how evaluations are conducted.
Learning & Growing through Evaluation: Briefs. https://www.cdc.gov/national-asthma-control-program/php/program_eval/briefs.html	These tools provide quick overviews on important evaluation topics: The *Enhanced Evaluation Framework* brief concisely describes the four how’s of practice detailed in the e-text. *Foundational Documents for the Program Evaluation Field* introduces people new to evaluation to the four foundational documents that provide guidance to the program evaluation field on how to evaluate programs well and ethically: The American Evaluation Association’s (AEA) Guiding Principles for Evaluators, The Joint Committee for Standards in Educational Evaluation’s program evaluation standards, AEA’s Public Statement on Cultural Competence in Evaluation, and AEA’s evaluator competencies. *Performance Measurement & Program Evaluation: A Suite of Evaluative Insights* helps new and seasoned evaluators to better understand how performance measurement and program evaluation are related. The brief provides insights about the usefulness of these inquiry methods and describes how they complement one another.

Abbreviation: CDC, Centers for disease Control and Prevention.

a At the time of this publication, all materials are available through asthma evaluation website (https://www.cdc.gov/national-asthma-control-program/php/program_eval/index.html). If you have any issues reaching the materials or would like to provide questions or feedback on specific materials, please contact the corresponding author, Samuel Dunklin at qaf3@cdc.gov.

## Tools for Learning and Growing Through Evaluation

Initially, NACP evaluation team members, dubbed evaluation technical advisors (ETAs), set out to develop a user-friendly guide that would walk novice evaluators through the full evaluation process; they would learn evaluation — build their evaluation capacity — as they used the tools. To ensure relevance, we worked with asthma program partners who served as advisors and reviewers, a process we continue. We called the guide, which evolved into a series of modules, *Learning and Growing Through Evaluation.* The title was intended to reinforce the idea that evaluation is a tool that can identify a program’s strengths and areas for growth.

We published the first module of *Learning and Growing Through Evaluation* in 2009, at the start of a new cooperative agreement (14,15). Module 1 introduced the concept of strategic evaluation planning, that is, working with interest holders to anticipate information needs throughout the 5-year cooperative agreement and creating a comprehensive portfolio of evaluations to meet those needs. At the time, longer-term evaluation planning like this was uncommon in the field, so we created a strategic evaluation plan (SEP) template that included fill-in-the-blank sections along with guidance on how to complete the plan. For example, one key task in creating a SEP is prioritizing evaluation investments among the many potential program elements that could be evaluated. The template aligns with sections in the module that describe various prioritization methods and offers sample criteria, like cost and equity.

The SEP template includes a timeline that encourages interest holders to map when evaluation findings will be needed against when they will be available. This can avoid timing missteps, like wrapping up an evaluation a month after a related grant application is due or starting to think about an evaluation near the end of a grant cycle, long past when relevant people and information are available. The timeline facilitates a cross-evaluation strategy whereby data collected in one evaluation might be leveraged in another. Finally, the SEP timeline helps program developers plan their evaluation capacity-building activities, looking ahead at evaluation needs to ensure that appropriate staffing or staff training are available when needed.

The first module also provides an evaluation plan template and guidance for evaluating the activities that were identified in the SEP as warranting evaluation. Unlike many evaluation plan templates that cover little more than data collection and analysis, the individual evaluation plan template lays out a blueprint for implementing all 6 steps of an evaluation. It starts with an interest holder assessment and engagement table and ends with guidance on documenting the evaluation’s implementation and acknowledging contributors.

The second *Learning and Growing* module moves beyond planning to provide strategies and tools for implementing and managing evaluations (16). It offers tips on addressing common challenges such as budgeting, and it introduces an action plan template that documents strategies for responding to an evaluation’s finding. We tailored subsequent modules to the strategies and activities asthma programs were using in their work, such as partnerships (17) and surveillance (18). The sixth and most recent module covers economic evaluation (19). Each module contains relevant tools and examples to facilitate evaluation processes while building evaluation capacity.

Early on, we were fortunate to collaborate with the US Environmental Protection Agency to develop a series of training webinars (20) that partners could access anytime, providing an avenue for continuous and sustainable engagement with the steps of the evaluation framework. The webinars range in length from 15 to 65 minutes, and some of the shorter webinars are ideal for funding recipients to share with their partners who need only a brief introduction to evaluation. Although the webinars provided general overviews of evaluation, we saw a need to understand and develop materials tailored to partner capacity.

With ETAs participating in monthly calls with partners, we had some insight into the types of evaluation activities partners were conducting, which in turn informed our materials development. To formalize this process, we created an evaluator self-assessment (21). ETAs and evaluators working in funded programs completed the self-assessment and flagged areas where professional development and additional tools would be helpful. A benefit of the assessment was that it highlighted instances in which novice staff members already possessed important skills, adding to their confidence in a new role. We also discovered the wealth of expertise among our partners, allowing us to draw on them as teachers for our community of practice.

One of the primary ETA roles is to review our partners’ evaluation plans — to add an external perspective to that of interest holders closely connected to the program. We discovered that evaluators had a difficult time understanding the differences among evaluation questions, research questions, and survey questions. We searched the evaluation literature and compiled an initial list of the characteristics of good evaluation questions, that is, questions that are likely to produce useful information. We then workshopped the list with several groups of experienced evaluators to establish agreed-upon criteria. From this, we created the Good Evaluation Questions Checklist (22). Evaluators and interest holders can use the checklist to sharpen the focus of their evaluations, review the evaluation’s standards, and document the rationale for their planning decisions.

Next, we took on the challenge of translating the literature on culturally responsive evaluation, which is “a holistic framework for centering evaluation in culture” (23). We partnered with CDC’s Division for Heart Disease and Stroke Prevention to create the guide, *Practical Strategies for Culturally Competent Evaluation* (24), and an accompanying tip sheet (25). The tools support evaluators in responding to the specific cultural contexts in which programs are working. Practicing evaluation in a culturally responsive way is important on principle and, instrumentally, it improves the validity of evaluative inferences. The Cultural Competence Assessment Tool (CCAT) (26) is a related tool that helps staff in state and local health departments assess their capacity to appropriately apply the US Department of Health and Human Services’ Culturally and Linguistically Appropriate Services standards (27).

One of our current focuses is on producing evaluation briefs (28–30), which provide short overviews of common evaluation topics. For example, when CDC added a performance monitoring component to its cooperative agreements, we noticed that partners had a hard time understanding how evaluation and performance monitoring work together. In response, we created a 4-page primer on the topic. Another current focus has been the co-development of an evaluation training series based on our materials in conjunction with the Climate and Health Program.

## Moving Upstream

To date, the primary audience for our resources has been practicing public health professionals. Our tools are designed to be practical: to enable anyone to competently perform a wide range of technical evaluative tasks and to give them tools for understanding and responding to the often-political contexts in which evaluations occur. In 2019, when we set out to update our *Learning and Growing through Evaluation* modules, we saw the opportunity to augment our materials with the theoretical and conceptual foundations of the evaluation field, to create a tool that would fill that gap in many public health professionals’ academic training. To this end, we published a free online evaluation textbook, *Planting the Seeds for High-Quality Program Evaluation in Public Health* (31).

The e-text marries theory and practice in a way that practitioners can easily apply. Though it is suitable for use in undergraduate and graduate courses, each chapter is informative for many audiences, from novice to advanced. Chapters end with review questions and skill-building exercises, and they include many of the tools and templates introduced in the modules. After reading the e-text, people who are brand new to evaluation will have a solid foundation for practice. Those who are more advanced will acquire skills and knowledge about evaluation approaches and techniques that they likely have not encountered. Our goal was to empower readers to have a better understanding of what is entailed in carrying out high-quality evaluations and what they can do to support and sustain high-quality evaluation practices in their organizations.

In creating the e-text, we recognized the need to update the original CDC Framework to include important advances in the discipline of evaluation. In our enhanced evaluation framework (Figure) we surrounded the steps and standards from the original framework with the characteristics that are important for evaluators to embody as they carry out their work: critical reflection, cultural responsiveness, situational awareness, and interpersonal competence. As evaluators, our ability to be aware of ourselves, others, and the broader environments in which we are working — the *how* of our practice — is equally as important as the technical steps we take. Our enhanced framework also recognizes the need to assess context (adding a step 0) and build evaluation capacity to ensure all interest holders can equitably engage in the evaluation process.

**Figure:**
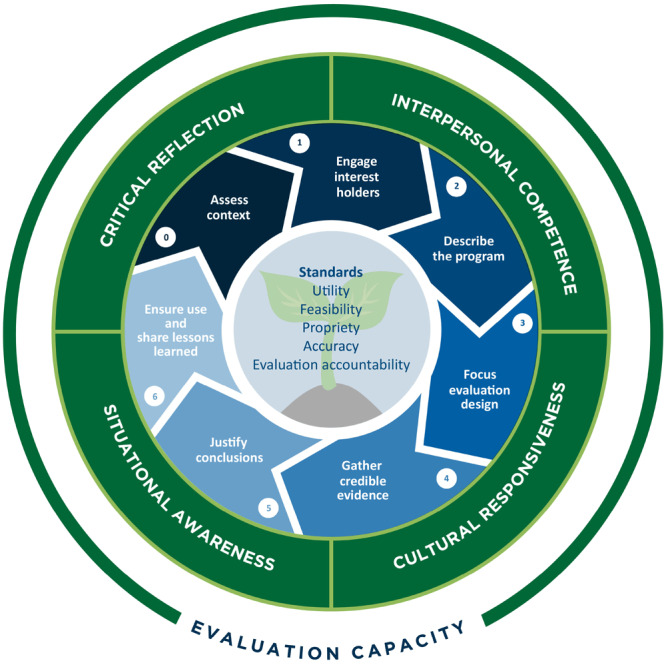
Enhanced evaluation framework.

Our enhanced framework resonated with many participants in a session at the 2022 AEA conference. Participants contributed their strategies and suggestions for implementing the 4 *hows* of evaluation practice (Table 2). As CDC’s Framework did in 1999, the e-text and our enhanced framework establish a new stepping-off point for using evaluation as a tool to make our public health programs more effective and equitable.

**Table 2:** Strategies to Implement the How’s of the Enhanced Evaluation Framework

How	**Strategies to Implement**
Interpersonal competence is a set of social skills for constructive interactions during the evaluation process, including communication, conflict resolution, and facilitation skills.	Establish trust Understand power and privilege in context Understand how power and privilege affect an evaluation Address conflicts Facilitate difficult conversations Help partners to articulate their views and understand other points of view Guide shared problem-solving and consensus building
Cultural responsiveness acknowledges and gives attention to the values, beliefs, and customs of a particular group or community. In an evaluation, cultural responsiveness means attending to the cultural aspects of a program and its interest holders in a respectful way while also being aware of one’s own cultural identity.	Be adaptable Create a diverse evaluation team: intersectionality of identities/cultures, lived experiences, different worldviews Encourage programs to include program participants and their families on their advisory board Understand the cultural values of interest holders, value co-creation Learn and appreciate each program’s cultural context and acknowledge that we may view and interpret the world differently from many evaluation interest holders Question organizational practices that do not necessarily work for all
Situational awareness is the ability of an evaluator to understand how contextual factors such as the program’s history, size, and complexity; the purpose of the evaluation (eg, formative, summative); evaluator experience, resource constraints; politics; and other factors affect evaluation design and use. Being situationally aware enables an evaluator to adapt and respond to these contextual factors by negotiating and implementing an evaluation that fits the intended uses.	Scan and assess context at regular intervals Anticipate need to change and stay flexible Accept and plan for leadership and staff changes Maintain strong partner relationships and check-ins
Critical reflection involves a “sustained and intentional process of identifying and checking the accuracy and validity” of one’s assumptions about their knowledge, values, beliefs, interpretations.	Take time before, during, and after a project to reflect on my role and identity, biases toward or within that project Talk openly and learn what shapes other’s views Collaborate with an external evaluator with different perspectives Walk and talk with friend whom you can trust to provide critical feedback, often called a critical friend Invite other perspectives from those with lived experience, end users, etc., on data analysis/sense-making

## Evidence of growth

Although we conducted an evaluation of our tailored technical assistance, we have not formally evaluated our collection of resources. Nevertheless, we have seen evidence of their influence among our partners in funded asthma programs and in the broader evaluation field. From the outset, our materials have framed evaluation as an opportunity to learn and grow rather than as a compliance activity done to meet a funder requirement. The response from our partners suggests that they see utility in this approach. In the spirit of learning, partners regularly contribute to AEA365, AEA’s blog; they often share their work at AEA’s annual meeting; and 4 state asthma programs voluntarily participated in a study on evaluative thinking, which was featured in *New Directions for Evaluation*, one of AEA’s flagship journals (32). And even though CDC’s cooperative agreements no longer include staffing requirements, most, if not all, funded partners dedicate a portion of their funding awards to support evaluation staffing.

NACP partners are applying what they learn to improve their programs. According to performance monitoring data NACP collected for its 5-year cooperative agreement that ended in 2019, partners took 426 actions based on their evaluation findings. Almost half of these actions related to improving, expanding, or sustaining specific interventions; other actions related to improving program infrastructure (surveillance, partnerships). We have published stories about our partners’ evaluation work in a document called *Learning as We Grow* (33,34); it is both a celebration of their work and a guide for others who are building out their evaluation capacity.

The reach of our materials has extended beyond the NACP. For example, in 2021, Thomas and Campbell included our Checklist for Assessing Your Evaluation Questions in their evaluation textbook, *Evaluation in Today’s World* (35); the text also referenced *Practical Strategies for Culturally Competent Evaluation.* Lovato and Hutchinson link to our webinars in their *Evaluation for Leaders* course (36). We have also received informal feedback from people who have put our tools to the test. Our materials have been called user friendly, concise, and accessible, and, in an especially gratifying email from a funded partner, they were described as “an exemplary demonstration of how to put the power of evaluation in the hands of people doing essential work within state health departments, and beyond.”

## Challenges and looking forward

Our tools are designed and vetted to ensure that they are user friendly and make evaluation approachable; however, even with these supports, evaluation can be challenging. The participatory approach fundamental to our enhanced framework takes time. Evaluation interest holders must be engaged and committed to a learning process. The day-to-day demands of implementing programs often seem to leave little time for conducting high-quality evaluations and acting on their findings.

We emphasize that evaluation is a tool for learning and growing, and still it can be difficult to dispel the notion that evaluation is a compliance activity, or an activity designed only to expose flaws rather than program strengths. Our hope is that as our partners and others in public health use our tools, they will develop an appreciative lens, seeing the assets and potential their programs possess. We hope they will see evaluation as a useful and grounding tool, especially during public health emergencies like the COVID-19 pandemic.

The tools developed and highlighted here will continue to be free and publicly available, serving as resources and guides to bolster evaluation practice. As evaluators in the NACP, we will continue to listen, respond, and adapt. We will continue to shine the light on evaluation as a tool to “make visible oppression and possibility,” in the words of evaluation scholar Donna Mertens (37). Evaluation is not a solo task. It is a difficult and time-consuming endeavor, a rewarding endeavor, that requires people from all walks of life to come together. As we look toward the future, we will be forever grateful for the colleagues who have, and who will, continue to learn and grow with us.
